# Treatment Outcome of Children with Retinoblastoma in a Tertiary Care Referral Hospital in Indonesia

**DOI:** 10.31557/APJCP.2021.22.5.1613

**Published:** 2021-05

**Authors:** Krisna Handayani, Braghmandita W Indraswari, Mei N Sitaresmi, Sri Mulatsih, Pudjo H Widjajanta, Wijnanda A Kors, Gertjan JL Kaspers, Saskia Mostert

**Affiliations:** 1 *Emma’s Children’s Hospital, Amsterdam UMC, Vrije Universiteit, Cancer Center Amsterdam, the Netherlands. *; 2 *Pediatrics, Faculty Medicine, Public Health and Nursing, Universitas Gadjah Mada, Dr Sardjito Hospital, Yogyakarta, Indonesia. *; 3 *Princess Máxima Center for Pediatric Oncology, Utrecht, the Netherlands. *

**Keywords:** Retinoblastoma, treatment outcome, low and middle-income countries

## Abstract

**Background:**

Although survival rates for retinoblastoma (RB) are over 95% in high-income countries, its high mortality rate in low and middle-income countries remains a great concern. Few studies investigated treatment outcome and factors contributing to RB survival in these latter settings. Aims of this study are to determine treatment outcome of Indonesian children diagnosed with RB and to explore factors predictive of treatment outcome.

**Methods:**

This study was a retrospective medical records review combined with an illustrative case report. Children newly diagnosed with RB between January 2011 and December 2016 at a tertiary care referral hospital in Indonesia were included. A home visit was conducted to perform an in-depth interview with a mother of two children affected by RB.

**Results:**

Of all 61 children with RB, 39% abandoned treatment, 21% died, 20% had progressive or relapsed disease and 20% event-free survival. Progressive or relapsed disease was more common in older (≥ 2 years at diagnosis, 29%) than young (<2 years at diagnosis, 0%) children (P=0.012). Event-free survival estimate at 5 years was higher in young (42%) than older (6%) children (P=0.045). Odds-ratio for event-free survival was 6.9 (95% CI: 1.747 – 27.328, P=0.006) for young versus older children. Other clinical and socio-demographic characteristics had no significant correlation with treatment outcome or event-free survival. The case report elucidated conditions and obstacles that Indonesian families face when their children are diagnosed with RB.

**Conclusion:**

Survival of children with RB in Indonesia is much lower compared to high-income and many other low and middle-income countries. Abandonment of treatment is the most common cause of treatment failure. Older age at diagnosis is associated with more progressive or relapsed disease and worse survival. Interventions to improve general public and health-care providers’ awareness, early detection and treatment adherence are required.

## Introduction

Retinoblastoma (RB) is the most common intraocular malignancy of childhood. Most children are younger than 5 years old at diagnosis, and the average age at diagnosis is 2 years. Retinoblastoma is potentially curable (Mostert et al., 2011; Singh and Daniels, 2016; Skalet et al., 2018; Thompson et al., 2015). Its prognosis depends on early diagnosis and appropriate therapy. Excellent survival has been reported in high-income countries. The five year survival rate in high-income countries is over 95%, but often less than 40% in low and middle-income countries. Problems and priorities differ between high-income versus low and middle-income countries. Whereas the goals of treatment in high-income countries have shifted towards globe or vision salvage and quality of life improvement, low and middle-income countries still focus on saving lives (Cassoux et al., 2017; Fernandes et al., 2017; Gao et al., 2016; Naseripour, 2012; Singh and Daniels, 2016). In the latter countries, the high mortality rate associated with RB is still of great concern (Jain et al., 2018; Naseripour, 2012). 

Factors that contribute to poor RB survival in low and middle-income countries include lack of awareness and knowledge of signs and symptoms of RB among the general public and among health-care providers. The subsequent delayed presentation, postponed referral to scarce specialized centers, late diagnosis and start of treatment enable the disease to get more advanced and reduce the chances for cure (Bai et al., 2011; Canturk et al., 2010; Cassoux et al., 2017; Chantada et al., 2011; Chawla et al., 2016; Chintagumpala et al., 2007; Jain et al., 2018; Leander et al., 2007; Miranda and Simanjutak, 2018; Naseripour, 2012; Pandey, 2014; Singh and Daniels, 2016; Sitorus et al., 2009). Socio-economic and cultural aspects, including families’ health beliefs about the disease and its curability, further deteriorate treatment adherence and survival (Leander et al., 2007; Mattosinho et al., 2016).

Understanding the clinical presentation and treatment outcomes of RB in low and middle-income countries is important in identifying the gaps and potential areas of RB treatment improvement. However, scarce scientific literature exists from most of these countries. 

The objectives of this study are: 1) to determine the treatment outcome of children diagnosed with RB at Dr Sardjito Hospital between 2011 and 2016, and 2) to explore factors predictive of treatment outcome. 

## Materials and Methods

*Setting*

Indonesia is a lower middle-income country with 262 million inhabitants. According to estimates of Indonesia’s age structure, 25 percent of Indonesians are under 15 years of age. Ten percent of the population lives below the poverty line (Central Intelligence Agency, 2016).

Dr Sardjito Hospital is a tertiary care referral and academic hospital located in Yogyakarta, central Java. The hospital is serving an estimated population of 5.8 million of whom 1.3 million children are below 15 years of age, and 430.000 children below 5 years (BPS-Statistik Indonesia, 2017). An estimated 150 children are newly diagnosed with cancer per year at our hospital, of which around 10 children have RB. This implies that the reported RB incidence of 10 cases is lower than the expected RB incidence (1:17,000) of 76 cases (World Health Organization (WHO), 2014). The pediatric oncology ward covers 41 beds and is operated by 4 pediatric oncologists and 31 pediatric nurses. Four types of wards are distinguished: VIP, 1st , 2nd, and 3rd class. The 3rd class ward has 23 of all 41 beds. Modality of RB staging and treatment was still limited at the time. Enucleation and chemotherapy were standard treatment. Radiotherapy availability was restricted, and laser/thermotherapy or cryotherapy was absent. Intraocular RB is treated by VEC Protocol, implying primary enucleation followed by six cycles of post-operative chemotherapy (vincristine, etoposide, carboplatin). Regional extension of RB is treated by Retinoblastoma 2002 GA II/III Protocol, and distant metastatic RB is treated by Retinoblastoma 2002 GA IV Protocol. Both protocols contain primary enucleation followed by 105 weeks of chemotherapy. Retinoblastoma 2002 GA II/III protocol consists of vincristine, methotrexate, doxorubicin, cyclophosphamide, whereas the Retinoblastoma 2002 GA IV Protocol adds cytarabine and etoposide as well.

*Study Design*

This study combines a retrospective medical records study with an illustrative case report. The investigation was conducted at Dr Sardjito Hospital. Inclusion criteria for the retrospective study were: 1) All children with newly diagnosed RB between January 2011 and December 2016; and 2) Age between 0-18 years at diagnosis.

Diagnosis of RB was based on clinical findings and ultrasonography, and confirmed by histopathology. CT-scanning was performed in patients with clinically obvious extraocular extension (proptosis). Both eyes were examined.

The following clinical and socio-demographic variables were collected from medical records: name, date of birth, gender, registration number of childhood cancer patient, date of first hospital admission, date onset of symptoms, type of first presenting symptoms, date of diagnosis, diagnosis delay, age at diagnosis, tumor extent (intra-ocular/ extra-ocular), stage of RB at diagnosis (low/ high), laterality (unilateral/ bilateral), date of start treatment, modality of treatment (enucleation and chemotherapy/ enucleation/ chemotherapy), parental socio-economic status, parental educational level, patients’ health-insurance status, distance to hospital, and treatment outcome. 

For age at diagnosis a cut-off point of 2 years was used, because previous studies showed that children who are older than 2 years at diagnosis have poorer outcome (Chawla et al., 2016; Fabian et al., 2020; Mattosinho et al., 2019; Rodrigues et al., 2004).

Parental socio-economic status divided children as coming from poor or prosperous families based on the assigned hospital class at diagnosis. Patients attending VIP, first and second class were classified as prosperous. Patients attending third class were classified as poor.

Children were classiﬁed as coming from families with low or high parental education. The parent with highest educational level determines designated level. Families with low parental education consist of families with parents with no education or with primary school and junior high school (implying ≤ 9 years education). Families with high parental education consist of families with a parent who was educated in senior high school, college or university (implying > 9 years education).

Health-insurance status was determined by whether patients did or did not have health-insurance at time of diagnosis. This data is routinely recorded in patients’ medical records at Dr. Sardjito Hospital.

The used stage classification was based on the International Retinoblastoma Staging System (IRSS) that divides RB in stage 0-IV. Staging systems are often adjusted to local settings. Our study divided RB in low stage (stage 0-I), intermediate stage (stage II-III) and high stage (stage IV) at diagnosis.(Chantada et al., 2006; Fabian et al., 2018) In case the patient’s medical record did not report a clear stage of disease at diagnosis, stage was based on assigned protocol. Patients with VEC Protocol were categorized as low stage. Patients with Retinoblastoma 2002 GA II/III Protocol were categorized as intermediate stage, and patients with Retinoblastoma 2002 GA IV Protocol as high stage. 

Diagnosis delay was defined as time between onset of signs or symptoms to confirmation of diagnosis. A cut-off point of 6 months was used, because previous studies showed that a lag time of more than 6 months was a prognostic factor for advanced RB (such as extraocular disease and metastasis) and poor outcome (Bai et al., 2011; Chawla et al., 2016; Mattosinho et al., 2019; Rodrigues et al., 2004).

Treatment outcome was defined as either first treatment failure that occurred (abandonment of treatment, death, progressive or relapsed disease), or in case no treatment failure occurred as event-free survival. In line with SIOP recommendations(Mostert et al., 2011), abandonment of treatment is defined as failure to start or continue scheduled curative treatment during 4 or more consecutive weeks.

A case study was performed in follow-up to the retrospective analysis to elucidate the conditions and challenges faced by some families of childhood RB patients. The investigators identiﬁed a family with two children diagnosed with RB. A home visit was conducted in September 2019 to interview the mother. Independent interviewers used a semi-structured questionnaire. Informed consent was obtained. The case report subsequently was checked and endorsed by the family concerned. 

The study was approved by Research Ethics Committee of Faculty Medicine, Public Health and Nursing, Universitas Gadjah Mada.

*Data Analysis*

Data management and analysis was done using SPSS version 22. The difference in socio-demographic and clinical patient characteristics and treatment outcome were analysed by chi-square and Fisher’s exact test. Kaplan-Meier survival analysis was carried out to compare the survival outcome between subgroups with different patient characteristics, using log-rank test. Event-free survival was measured from the date when the patient received a diagnosis of RB to the first treatment failure (abandonment of treatment, death, progressive or relapsed disease) or the date of last follow-up. Overall survival was measured from the date when patient received diagnosis to the date of death or the date of last follow-up. Univariate and multivariate backward stepwise logistic regression analysis evaluated the relationship between patient characteristics and event-free survival. A 2-sided P value of less than 0.05 was considered statistically significant.

## Results

*Retrospective Medical Records Study*

Between January 2011 and December 2016, 61 children were newly diagnosed with RB. The age at diagnosis ranged between 3 and 130 months old with a mean of 33 months and median of 28 months. Table 1 shows socio-demographic and clinical patient characteristics. Most RB patients (n=42, 89%) were referred to Dr Sardjito Hospital from other facilities at: secondary (95%) and tertiary (5%) care level. Before coming to Dr. Sardjito Hospital, 29 patients (48%) had already been diagnosed with RB, and 8 patients (13%) already underwent enucleation. At Dr. Sardjito Hospital patients were initially admitted to: eye department (n=29, 48%), pediatric department (n=18, 30%), unclear (n=14, 23%). Familial history of RB was only documented and confirmed in medical records of 2 patients (3%). All other medical records had no notification about its presence or absence.

Patients presented with following symptoms: leukocoria/ cat eye (86%), red eye (30%), proptosis (25%), lacrimation (14%), vision reduction (14%), ocular pain (11%), and strabismus/squint (9%). Tumor extent (intra-ocular/ extra-ocular) was not accurately documented in medical records and therefore could not be retrieved. At diagnosis, cancer was classified as: unilateral (77%) or bilateral (23%). Diagnosis delay ranged between 0 and 60 months, with median of 12 months. In total, 67% of patients had diagnosis delay of more than 6 months. 

Stage of RB at diagnosis could only be abstracted from medical records of 50 (82%) patients: low stage (2%), intermediate stage (42%), and high stage (56%). It is noteworthy that the single patient with low stage RB had stage I. The stage of disease was clearly documented in medical records of 19 patients (38%), yet had to be based on assigned protocol in 31 patients (62%). The remaining 11 (18%) patients had no recorded staging. 

Provided treatment was only documented in medical records of 46 (75%) patients: enucleation and chemotherapy (65%), chemotherapy (24%), and enucleation (11%). Used chemotherapeutic protocol was only recorded in the medical records of 34 (56%) patients: VEC Protocol (6%), Retinoblastoma 2002 GA II/III Protocol (44%), Retinoblastoma 2002 GA IV Protocol (50%).

**Figure 1:**
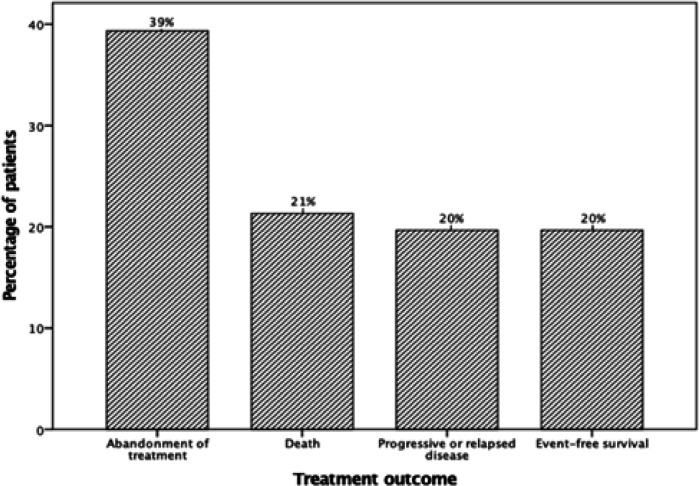
Treatment Outcome of Children with retinoblastoma (n=61)

**Figure 2:**
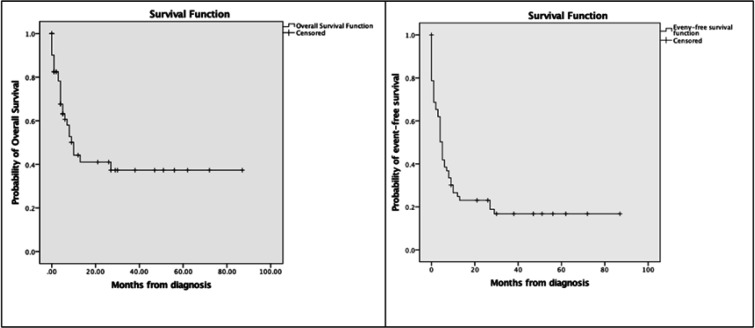
Kaplan-Meier Estimates of Overall Survival and Event-Free Survival of Children with Retinoblastoma (n=61). Events included abandonment of treatment, death, and progressive or relapsed disease

Figure 1 presents treatment outcome of children with RB. Most common treatment failure was abandonment of treatment (n=24, 39%): 29% of patients abandoned before start of treatment, and 71% during treatment. Death was second most common cause of treatment failure (n=13, 21%): treatment-related (62%), and cancer-related (38%). Treatment-related death causes were infection, sepsis and septic shock. Cancer-related death cause was intracranial metastasis. Least common treatment failure was progressive or relapsed disease (n=12, 20%): progressive disease (33%), and relapse (67%). In total, 12 patients (20%) had event-free survival. Figure 2 illustrates that overall survival estimate at 5 years after diagnosis was 37%, and event-free survival estimate at 5 years after diagnosis was 17%. Note that Figure 1 presents actual percentages, whereas Figure 2 depicts time-dependent probability estimates. 

**Figure 3:**
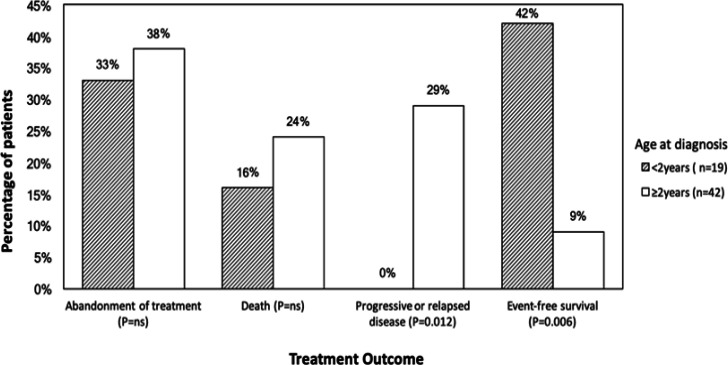
Treatment Outcome of Children with Retinoblastoma Per Age at Diagnosis (n=61)

**Figure 4:**
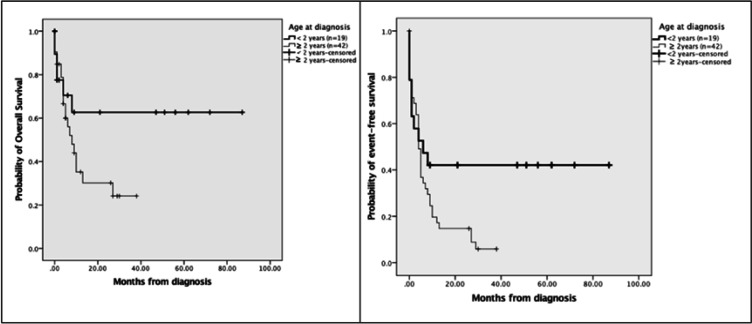
Kaplan-Meier Estimates of Overall Survival (P=Ns) and Event-Free Survival Per Age at Diagnosis (P=0.045).Events included abandonment of treatment, death, and progressive or relapsed disease. Heavy solid line, patients with age at diagnosis <2 years (n=19); solid line, patients with age at diagnosis ≥ 2years (n=42)

Of 61 patients with RB, 19 patients (31%) were young (< 2 years), and 42 patients (69%) were older (≥ 2 years) at diagnosis. Long diagnosis delay (> 6 months) was seen in more older (80%) than young (40%) children (P=0.019). Figure 3 shows that treatment outcome differed significantly between young and older children. Progressive or relapsed disease was more common in older (29%) than young (0%) children (P=0.012), whereas event-free survival occurred more in young (42%) than older (9%) children (P=0.006). The odds-ratio for event-free survival was 6.9 (95% CI: 1.747 – 27.328, P=0.006) for young versus older children. Figure 4 shows that overall survival estimate at 5 years after diagnosis was 63% for young versus 24% for older children (P=Ns), and that event-free survival estimate at 5 years after diagnosis was 42% for young versus 6% for older children (P=0.045) 

The other socio-demographic (distance to hospital, parental socio-economic status, parental educational level, health-insurance status) and clinical (gender, laterality, stage of disease at diagnosis, modality of treatment, diagnosis delay) patient characteristics had no significant impact on treatment outcome, overall survival estimates and event-free survival estimates. 

*Case Report*

When their first daughter was 7 months old, the mother noticed that her daughter’s eye glinted when exposed to light. She brought her daughter to a primary care facility where the attending nurse immediately referred them to Dr Sardjito Hospital. At the eye department, the ophthalmologist explained the parents about RB and indicated that the child required surgery and chemotherapy. However, the parents had difficulties to comprehend provided information. There was no family history of RB and the parents had never heard of this disease before. They were not aware that their daughter’s intraocular RB had good prognosis if they would start treatment promptly. The ophthalmologist referred them to the pediatric hematology-oncology division for further investigations and treatment. However, the parents believed that their daughters’ disease could not be cured and that her fate was in God’s hands. The parents also feared surgery and felt that their daughter was too young to be operated on. Because the family had no health-insurance and only the father had an irregular income as farm worker, they could not afford prescribed expensive treatment at Dr Sardjito Hospital. Therefore, the parents decided to abandon conventional cancer treatment and sought complementary alternative medicine instead. But when their daughter was 2.5 years old, the parents brought her back to Dr Sardjito Hospital, because the mass had become bigger and now both eyes proptosed. Due to the girl’s poor condition, no curative treatment was given. She passed away after 22 days of hospital care. The mother expressed that these memories were so painful that she had thrown away pictures and medical reports of her first daughter. 

Dissimilar with experiences in their first daughter, the mother immediately recognized the signs of RB in their second daughter when she was 2 months old: her eye reflected light when exposed to flash light. After promptly attending a primary care facility, the family was referred to Dr Sardjito Hospital. This time the parents, having learned from their previous experience, agreed with conventional cancer treatment. They no longer feared surgery and were convinced that their daughters’ life could be saved if there would be no delay in diagnosis and start of treatment. The family was able to obtain government-subsidized health-insurance for people living below the poverty line, which covered both diagnostics and treatment. After the diagnosis of intraocular unilateral RB was confirmed, the second daughter received enucleation, post-operative chemotherapy, and prosthesis for her eye. After successful completion of conventional cancer treatment, the girl now returns to Dr Sardjito Hospital for follow up every 6 months. Hereditary cases often suffer from bilateral disease and have to be followed at least until the age of 5 years. She has recently turned 9 years old. The girl has good vision in her remaining eye and regained normal life, attends school and plays with friends.

According to the mother, the father blames his wife that she has caused RB in both their children due to a genetic disorder. The father has left the family and wants a divorce to marry another woman to have healthy children with. 

**Table 1:** Patients’ Socio-Demographic and Clinical Characteristics (n=61)

Characteristics	Number of Patients n (%)
Gender	
Male	34 (56)
Female	27 (44)
Age at diagnosis	
<2 years	19 (31)
≥2 years	42 (69)
Distance to hospital	
<50 km	13 (21)
50-100 km	29 (48)
>100 km	19 (31)
Parental socio-economic status (n=60)	
Poor	45 (75)
Prosperous	15 (25)
Parental educational level (n=54)	
Low	18 (30)
High	36 (60)
Health-insurance status	
No	34 (56)
Yes	27 (44)
Laterality (n=47)	
Unilateral	36 (77)
Bilateral	11 (23)
Tumor extent (n=43)	
Intraocular	18 (42)
Extraocular	25 (58)
Stage of disease at diagnosis (n=50)	
Low stage (stage 0-I)	1 (2)
Intermediate stage (stage II-III)	21 (42)
High stage (stage IV)	28 (56)
Modality of treatment (n=46)	
Enucleation and chemotherapy	30 (65)
Chemotherapy	11 (24)
Enucleation	5 (11)
Diagnosis delay (n=45)	
0-6 months	15 (33)
>6 months	30 (67)

## Discussion

Worldwide, RB is diagnosed in approximately 8,000 children per year. Of these children, 11% live in high-income countries, 69% in middle-income countries and 20% in low-income countries (Dimaras et al., 2015). Survival may be as high as 95% in high-income countries, but often is below 40% in low and middle-income countries. In our study the event-free survival estimate at 5 years of children with RB in Indonesia, a lower middle-income country, was only 17%. This is much lower than reported in other neighboring Asian countries, like India (65%) and Thailand (60%) where public awareness campaigns have successfully reduced diagnosis and treatment delay (Chawla et al., 2016; Gupta et al., 2019; Wongmas et al., 2015).

Challenges faced by low and middle-income countries that contribute to low RB survival include: limited awareness of RB among general public and health-care providers, lack of national screening programs, delayed seeking of medical attention, poor referral networks, scarcity of specialized treatment centers, unavailability of advanced diagnostic tools and treatment modalities, lacking prosthetic devices, and absence of efficient multidisciplinary team collaborations (Butros et al., 2002; Faranoush et al., 2014; Jain et al., 2018; Naseripour, 2012; Singh and Daniels, 2016). All these challenges contribute to advanced disease at diagnosis. A global review on RB presentation of 4,351 patients from 153 countries showed that at diagnosis stage 0 was present in 56.8% of RB patients in high-income countries, 48.8% of RB patients in upper middle-income countries, 24.2% in lower middle-income countries and 8.7% in low-income countries. And that children were diagnosed with stage IV in 0.5% of RB patients in high-income countries, 5% of RB patients in upper middle-income countries, 8.3% in lower middle-income countries and 18.7% in low-income countries (Fabian et al., 2020). By contrast, none of the Indonesian children in our study had stage 0 RB at diagnosis and an alarming 56% had stage IV. This emphasizes the urgent need for interventions to prevent diagnosis delays in Indonesia. Community awareness campaigns and training of personnel in primary health care facilities, like midwives, general practitioners and pediatricians need to be established, particularly in remote areas. For instance, routinely inspecting both eyes during each vaccination and provision of information leaflets to families could be very helpful. 

The most common reason for treatment failure in our study population was abandonment of treatment. This finding is in line with abandonment rates of 30-40% reported in other RB studies from low and middle-income countries, and contrasts the virtual absence of this phenomenon (1%) in high-income countries (Chantada et al., 2011). Psychosocial and economic factors influencing parental decision to allow or refuse potentially lifesaving enucleation in children with RB are health beliefs that cancer is a fatal illness, common cultural and religious beliefs, beauty expectations of particularly girls, the fear of unacceptable aesthetic outcome of surgery, extended family pressure to reject enucleation, and the cost of treatment. This makes adequate parental education and psychosocial guidance mandatory. Studies from India and the Philippines proof that effective counselling about enucleation can decline abandonment rates, despite financial problems of families.(Alcasabas et al., 2014; Kumar et al., 2013) However, in most low and middle-income countries parental education and psychosocial guidance are not prioritized (Butros et al., 2002; Domingo et al., 2017; Faranoush et al., 2014; Jain et al., 2018; Naseripour, 2012; Singh and Daniels, 2016). Also in our case report was found that the parents had difficulties to comprehend provided medical information. They were not aware initially that RB has a good prognosis if they would start treatment promptly. The parents believed that their first daughters’ disease could not be cured and that her fate was in God’s hands. The parents also feared surgery and felt that the girl was too young to be operated on. As the family lacked health-insurance, and only the father had an irregular income as farm worker, they could not pay the high treatment costs at Dr Sardjito Hospital. Consequently, the family decided to abandon conventional cancer treatment and sought complementary alternative medicine instead with their first affected child. When their second daughter got ill, the family registered for government-paid health-insurance for citizens living below the poverty line. This enabled access to adequate medical care and prevented long-term financial hardships. In addition, it underlines the importance of the recent implementation of Universal Health Coverage by the Indonesian government (Indraswari et al., 2021). 

Older age (≥ 2 years) at diagnosis was associated with more progressive or relapsed disease and worse survival. The median age at diagnosis in our study was 28 months. This finding is similar with other studies from Asia (Chang et al., 2006; Chawla et al., 2016; Gao et al., 2016; Kaliki et al., 2019; Subramaniam et al., 2014). The global review on RB presentation showed that the median age at diagnosis was 14.1 months in high-income countries, 25 months in upper middle-income countries, 29.5 months in lower middle-income countries, and 30 months in low-income countries (Fabian et al., 2020). Older age at diagnosis in low and middle-income countries reflects delayed seeking of medical help, postponed referral to specialized centers, and late diagnostics (Essuman et al., 2011; Handayani et al., 2016; Nyawira et al., 2013). In our study 67% of patients had a diagnosis delay of more than 6 months, and this longer delay was significantly more often seen in older children. Both parents and health-care providers do not recognize the early signs and symptoms of RB. In a previous study conducted at Dr Sardjito Hospital, we found that patient delay was significantly shorter than health-care system delay (Handayani et al., 2016). Availability of advanced diagnostic facilities may be lacking in many resource-poor settings delaying the establishment of diagnosis and staging. Even though diagnosis delay in our study did not significantly influence event-free survival, several other studies have shown that RB survival is higher among patients with lag times less than 6 months (Bai et al., 2011; Butros et al., 2002; Goddard et al., 1999; Rodrigues et al., 2004).

Particularly in resource-limited settings, preference of complementary alternative medicine and fear of RB surgery have been reported as reasons for postponed seeking of medical help, diagnosis and start of conventional cancer treatment (Chang et al., 2006). A prior study at Dr Sardjito Hospital indeed also confirmed that using alternative treatment was associated with significant longer patient and total delay (Handayani et al., 2016). This late presentation results in older age at diagnosis and poorer prognosis due to progression of disease and local or advanced metastasis (Gupta et al., 2019; Hu et al., 2018). Our case report illustrates this clearly. The parents feared enucleation in their first daughter and resorted to alternative medicine instead. By the time they turned to conventional medicine again, the disease had progressed and was no longer curable. 

A familial history of RB was only documented and confirmed in the medical records of 3% of patients in our study. The global review on RB presentation illustrated that a familial RB history is present in 8.4% of RB patients in high-income countries, 4.5% of RB patients in upper middle-income countries, 4% in lower middle-income countries and 3.1% in low-income countries. The limited number of familial cases in low and middle-income countries can partially be related to underreporting and lacking medical chart documentation. But more importantly, children with RB in high-income countries are diagnosed and treated promptly and therewith reach childbearing years, while most children in low and middle-income countries still come with advanced disease stages and die young (Fabian et al., 2020; Miranda and Simanjutak, 2018).

Many of our patients come from low socio-economic and educational backgrounds. Their families live in rural areas with predominant patriarchal cultures where traditional health beliefs regarding female fertility and stigmatization of “hereditary defects” are still strong. This is also illustrated by the father in our case report who blamed the mother for giving birth to children with “genetic abnormalities.” Prejudices against women with reproductive system problems are unfortunately still common in these rural societies, causing problems in their relation to men and often leading to separation and divorce (Pujiati, 2016; World Health Organization (WHO), n.d.). 

Main limitations in this study were small sample size and missing data due to the retrospective nature of the medical records study. We learnt that documentation of certain clinical characteristics, for example tumor location, stage of disease at diagnosis, laterality, family history, and applied chemotherapeutic protocol needs to be rigorously ameliorated. The poor state of medical records and missing staging and treatment data hinder getting a true analysis of the situation. Health professionals therefore need education sessions focusing on the importance of good record keeping and their responsibility to daily document their patients’ information in correct ways. In addition, the medical record department must improve its management. As the study took place in a single center, caution with generalizability of findings must be applied. Further research on a larger group of RB patients is needed before definitive conclusions regarding influence of socio-demographic and clinical characteristics on RB treatment outcome can be drawn. There is an urgent need to establish a national cancer registry. Collecting data of rare types of childhood cancer on a national scale is important for analysis and direction of future health policies. A strength of this study was the inclusion of the case report. The mother’s story clearly illustrated the conditions and obstacles that Indonesian families face when their children are diagnosed with RB. It painfully illustrates that if better information had been available, the parents might have consented with curative treatment of their first child too. 

In conclusion, survival of children with RB at Dr Sardjito Hospital in Indonesia is much lower compared to high-income and many other low and middle-income countries. Abandonment of treatment is the most common cause of treatment failure. Older age at diagnosis is associated with more progressive or relapsed disease and worse survival. Based on findings of this study, we recommend the following: a) Community awareness campaigns about early signs and symptoms of cancer should be organized by the government to stimulate families to immediately seek medical care; b) Training to improve recognition of cancer symptoms and fasten subsequent referral and diagnostic process is required for medical teams at primary, secondary, and tertiary health care facilities; c) Parental education programs and parent supportive meetings are required to better inform and guide families of children with RB and ensure their treatment adherence. These combined measures can limit arrears. When children with RB are referred and admitted faster with less-advanced stages of disease and better visual prognosis, this will eventually result in more children achieving cure from RB.

## Author Contribution Statement

MNS, GLK and SMT conceptualized and designed the study. KH, BWI, MNS and SMT conceptualized and designed the data collection instruments. SMH, MNS and SMT coordinated and supervised the data collection. KH and BWI collected the data. KH, BWI and SMT analyzed the data. All authors interpreted the data. KH drafted the initial manuscript. BWI, MNS, SMH, PHW, WAK, GLK and SMT critically reviewed and revised the article. All authors approved the final manuscript as submitted.
